# FROM FAILURE TO OPPORTUNITY IN QUEERING GERONTOLOGICAL CURRICULUM

**DOI:** 10.1093/geroni/igad104.0258

**Published:** 2023-12-21

**Authors:** Lauren Bouchard

**Affiliations:** Portland State University, Portland, Oregon, United States

## Abstract

Institutes of higher education vary tremendously in their support of LGBTQ students, which can contribute to unequal educational opportunities and outcomes. The presence of LGBTQ people in education can be inherently divisive, with some states and educational institutions prohibiting the inclusion of LGBTQ-related topics in the curriculum. LGBTQ students who experience discrimination in the classroom and heterosexism in the curriculum may be less motivated to complete program requirements, resulting in lower completion rates. Attrition of these marginalized students can lead to fewer qualified gerontologists who reflect the increasing diversity of the aging population. Additionally, the gerontological curriculum within and across programs may fail to capture nuanced and diverse narratives of resilience and lived experiences due to dominant, heteronormative educational contexts and frameworks. Through the lens of the queer art of failure, the presenter utilizes autoethnography (e.g., analysis of past writing and personal communications) as a queer person in a non-affirming gerontology graduate program. They will discuss how institutional failure lead to enthusiasm for curriculum development, student mentoring, and queer knowledge dissemination. Key findings include the significance of institutional clarity on academic freedom, the necessity of institutional support, and the solidarity of LGBTQ students, faculty, and older adults. The presenter will conclude with their future research directions and strategies to support students and early-career scholars to overcome political, institutional, and what are often felt as personal failures.

